# A 3D musculoskeletal model of the western lowland gorilla hind limb: moment arms and torque of the hip, knee and ankle

**DOI:** 10.1111/joa.12651

**Published:** 2017-07-17

**Authors:** Colleen Goh, Mary L. Blanchard, Robin H. Crompton, Michael M. Gunther, Sophie Macaulay, Karl T. Bates

**Affiliations:** ^1^ Department of Musculoskeletal Biology Institute of Aging and Chronic Disease University of Liverpool Liverpool UK; ^2^ School of Biosciences University of Birmingham Birmingham UK

**Keywords:** 3D modelling, adduction‐abduction, flexion‐extension, locomotion, moment arms, western lowland gorilla

## Abstract

Three‐dimensional musculoskeletal models have become increasingly common for investigating muscle moment arms in studies of vertebrate locomotion. In this study we present the first musculoskeletal model of a western lowland gorilla hind limb. Moment arms of individual muscles around the hip, knee and ankle were compared with previously published data derived from the experimental tendon travel method. Considerable differences were found which we attribute to the different methodologies in this specific case. In this instance, we argue that our 3D model provides more accurate and reliable moment arm data than previously published data on the gorilla because our model incorporates more detailed consideration of the 3D geometry of muscles and the geometric constraints that exist on their lines‐of‐action about limb joints. Our new data have led us to revaluate the previous conclusion that muscle moment arms in the gorilla hind limb are optimised for locomotion with crouched or flexed limb postures. Furthermore, we found that bipedalism and terrestrial quadrupedalism coincided more regularly with higher moment arms and torque around the hip, knee and ankle than did vertical climbing. This indicates that the ability of a gorilla to walk bipedally is not restricted by musculoskeletal adaptations for quadrupedalism and vertical climbing, at least in terms of moment arms and torque about hind limb joints.

## Introduction

Many extant primates have been regarded as adapted to a specialised mode of locomotion. For example, orang‐utans and gibbons are classically described as suspensory (Rose, 1988; Tuttle & Cortright, 1988; Hunt, 1991), and gorillas and chimpanzees as terrestrial knuckle‐walkers and vertical climbers (Hunt, 1991; Gebo, 1996; Remis, 1998). In many instances, these species have specific musculoskeletal adaptations to a predominant locomotor mode, such as a strongly developed flexor digitorum brevis that arises from both the medial calcaneal tubercle and plantar aponeurosis in gorillas, which is similar to that in humans and is argued to be associated with propulsion during terrestrial locomotion (Sarmiento, 1994; Kelikian & Sarrafian, 2011; Kulkarni, 2011). Such morphological adaptations are not only informative about living primates, but are often employed in interpreting the locomotor anatomy and ecology of extinct primates from fossil remains (Richmond et al. 1998; Crompton et al. 2008; DeSilva, 2009; Lovejoy et al. 2009). In particular, musculoskeletal adaptations for arboreal and terrestrial locomotion in living great apes have been extensively studied to understand the origin and evolution of bipedal locomotion in humans (Fleagle et al. 1981; Vereecke et al. 2005; Thorpe & Crompton, 2006; Thorpe et al. 2007a; Crompton et al. 2010; Bates et al. 2013).

Muscles generate the forces and powers required for movement. Moment arms represent one important aspect of muscle mechanics that can be readily measured in extant animals (Spoor & Van Leeuwen, 1992; Young et al. 1993; Miller & Dennis, 1996; Boyd & Ronsky, 1997; Thorpe et al. 1999; Arnold & Delp, 2000; Krevolin et al. 2004; Payne et al. 2006b; Channon et al. 2010; O'Neill et al. 2013; Hutchinson et al. 2014) and estimated in extinct taxa with near‐complete skeletons (Hutchinson et al. 2005; Bates & Schachner, 2012; Bates et al. 2012a,b; Maidment et al. 2014). The moment arm of a muscle quantitatively defines its leverage capacity relative to a joint and, more specifically, its ability to convert contraction force into rotational force (i.e. torque) at the joint centre (Zajac, 1992). Elastic contribution from tendons being equal, larger moment arms will result in higher joint torques or moments for a given muscle contraction force.

Geometric constraints on muscle paths mean that moment arms, and hence torque, can vary with joint angles. This has led to suggestions that animals will favour specific postures or ranges of joint angles during their habitual locomotor activities in which moment arms and/or torques are maximised (Payne et al. 2006b; Michilsens et al. 2010; Fujiwara & Hutchinson, 2012). Indeed, Michilsens et al. (2010) found that siamangs maximise moment arms around the elbow during the support phase of brachiation, and Fujiwara & Hutchinson (2012) found that relative elbow moment arms reliably indicate different limb postures in terrestrial quadrupeds. Payne et al. (2006b) suggested that higher extensor moment arms at flexed positions were linked to use of flexed postures during arboreal quadrupedalism and climbing in bonobos, western and eastern lowland gorillas, and gibbons. This latter finding is particularly interesting for a number of reasons. To our knowledge, no other study of muscle moment arms in terrestrial tetrapods has found whole‐scale stabilisation or increases in extensor (anti‐gravity) muscle moments and torques in flexed limb postures. Most, if not all, studies of moment arms in humans (Spoor & Van Leeuwen, 1992; Krevolin et al. 2004), non‐human primates (Ogihara et al. 2009; Channon et al. 2010; O'Neill et al. 2013), other mammals (e.g. horses; Brown et al. 2003), birds (Goetz et al. 2008; Hutchinson et al. 2014) and crocodilians (Bates & Schachner, 2012) report the tendency for extensor moment arms to decrease as joints become more flexed. That the opposite tendency was found for bonobos and western and eastern lowland gorillas might be held to imply that these species have unique morphological adaptations that maintain relatively high moment arms for extensor muscles in flexed postures. However, no such morphological explanations for extensor moment arm patterns were proposed by Payne et al. (2006b), nor to date has this novel finding been investigated further in bonobos and lowland gorillas. Payne et al. (2006b) highlighted the substantial intra‐individual variability in their data and note that their study ‘was performed on a small sample of apes and thus differences noted here warrant further investigation’ (Payne et al. 2006b, page 725).

In this study, we revisit hind limb muscle moment arms in the western lowland gorilla using a 3D musculoskeletal model, and make this model freely available for further research. The model is produced in the freely available multi‐body dynamics package gaitsym (www.animalsimulation.org) and is readily adaptable for a range of morpho‐functional investigations, as well as forward dynamics simulations (Sellers et al. 2003, 2004, 2010, 2013; Sellers & Manning, 2007). Herein, we use this model to estimate 3D muscle moment arms and, in conjunction with additional data on muscle geometry and architecture from dissections, address the following objectives:

1. Compare moment arm predictions from our 3D model to previously published data (Payne et al. 2006b) and discuss the implications of similarities and differences on interpretations of locomotion in western lowland gorillas.
2. Investigate whether joint angle ranges used for climbing, terrestrial quadrupedalism and bipedalism correspond to higher moment arms and torques.

## Material and methods

### Dissection

Anatomical dissection was carried out on a gorilla that was euthanised in a zoo on 5 October 2011 at 46 years, 8 months of age after suffering from age‐related pathologies. These contributed to significant weight loss just before her death, but keepers noted no change in gait (pers. comm. to C.G.). She weighed 72 kg at time of death and was stored in a freezer after necropsy. Her femur was 34 cm long (from most proximal point of femur head to most distal point of medial condyle). Her tibia was 30.7 cm long (from most proximal point of medial condyle to most distal point of medial malleolus) and fibula was 28.5 cm long (from most proximal point of fibula head to most distal point of lateral malleolus). All length measurements were made directly on the bones using a measuring tape, accurate to 0.01 m, after muscles were removed.

Muscles were identified with reference to Diogo et al. (2010). Where origins/insertions could reasonably be approximated to a centroid (e.g. semitendinosus), the location of this centroid point was recorded descriptively in relation to bony markers and measured (using a ruler) to determine how proximal/distal/medial/lateral it was to these markers. Where the origins/insertions were of a larger area (e.g. gluteus medius), the same method was used to record a selection of points defining the borders of the attachment area and additional qualitative descriptions were noted (e.g. relationships to bony landmarks and/or other muscles) alongside photographs for each muscle after separation from other muscles while still attached and after removal. As the gorilla used for creating the bones of the model was different to the one that was dissected (see below), the measurements taken were used as a guide along with photographs to link dissection data to the choice of attachment sites for the model. Abbreviations used for muscles are given in Table 1.

**Table 1:** List of muscles identified during dissection in alphabetical order, along with abbreviations in brackets, if any. Muscles where attachment was cut during necropsy or skinning are indicated

Abductor digiti minimi	Flexor hallucis longus (FHL)	Popliteus – cut at origin
Abductor hallucis	Gastrocnemius – cut at origin	Psoas major – cut at origin
Abductor metatarsi quinti	Gemellus inferior	Quadratus femoris
Adductor brevis	Gluteus maximus	Rectus femoris
Adductor hallucis brevis	Gluteus medius	Sartorius – cut at insertion
Adductor longus	Gluteus minimus	Semimembranosus – cut at insertion
Adductor magnus	Gracilis – cut at insertion	Semitendinosus – cut at insertion
Biceps femoris ‐ cut at insertion	Iliacus	Soleus
Extensor digitorum longus (EDL)	Iliocapsularis	Tibialis anterior
Extensor hallucis longus (EHL)	Ischiocondylica	Tibialis posterior
Fibularis brevis	Obturator externus	Vastus intermedius
Fibularis longus	Obturator internus	Vastus lateralis
Flexor digitorum longus (FDL)	Pectineus	Vastus medialis

Mass (using an Adam Equipment PGW 2502i lab balance electronic scale, accurate to 0.01 g) and length (using a ruler) were measured for each muscle, tendon, and muscle‐tendon unit. Fibre length measurements for each muscle were taken five times and the average calculated. Physiological cross‐sectional area (PCSA) is usually calculated as [muscle volume*cos(fibre pennation angle)]/fascicle length (FL). However, as fibre pennation angles for most mammal lower limb muscles are small enough (< 30°) that the effect on PCSA should be minimal (Thorpe et al. 1999; Carlson, 2006), we calculated PCSA by dividing muscle volume (measured mass/muscle density) by FL (Thorpe et al. 1999; Payne et al. 2006a). All muscle data, scaled to body mass of the gorilla from the CT scan (see below), can be found in the Supporting Information (Appendix S1, Figs S1–S2, Table S1).

### Building the 3D musculoskeletal model

#### The skeletal model

Existing CT data of a sub‐adult male western lowland gorilla weighing 152 kg at time of death was used as a basis for the musculoskeletal model, as the dissected gorilla (see above) was not suitable due to skeletal damage and partial dissection carried out by another researcher. The sub‐adult male gorilla was CT scanned at the University of Liverpool Small Animal Hospital using a Siemens Volume Zoom (4 slice) scanner. Using the same anatomical markers as in the dissected gorilla (see above), the femur was 27.1 cm, tibia 22.6 cm and fibula 20.9 cm long. A surface mesh of its left hind limb skeleton was created using amira 5.4.3. The computer‐aided design package maya (www.autodesk.com) was used digitally to rearticulate hind limb bones in a standard neutral posture and to rig 3D muscle–tendon units and joint centre positions as in previous studies (Bates & Schachner, 2012; Bates et al. 2012a,b, 2015; Maidment et al. 2014; see Supporting Information Appendix S1, Figs S1–S2, Table S1 and Video S1–S3 for further details). The final model was composed of the following 24 segments: trunk, thigh, shank, rear foot, and metatarsals, proximal phalanges, middle phalanges and distal phalanges one to five. In this study we only present muscle moment arms about the hip, knee and ankle. The segments within the foot were held fixed throughout and have no impact on the data presented herein.

**Figure 1:**
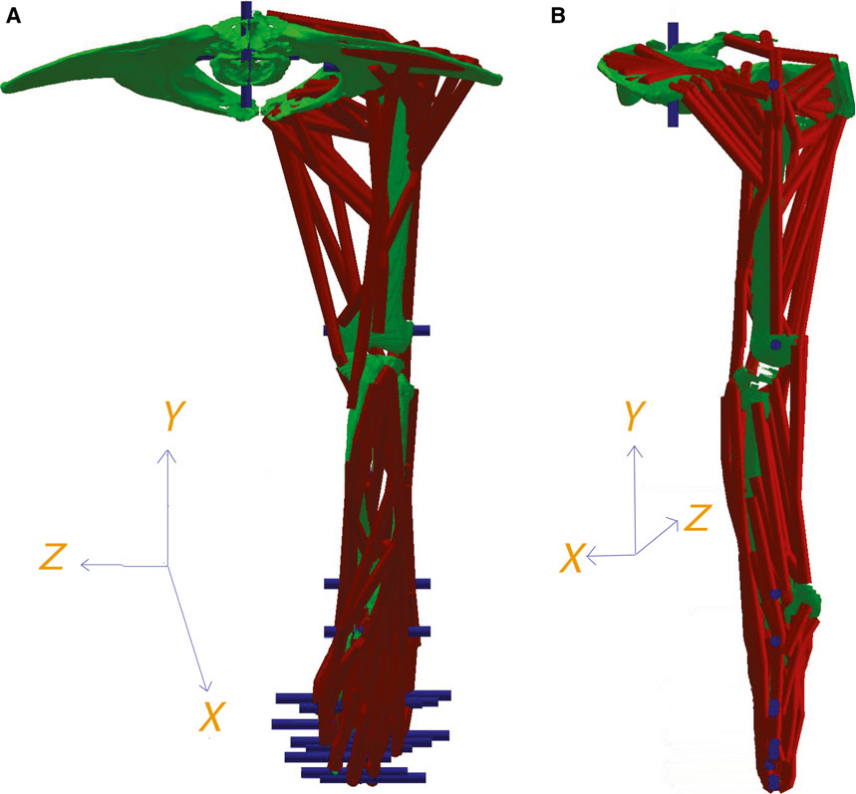
(A) Anterior and (B) lateral views of hind limb at neutral position. Muscle paths are red, joint axes are blue. Note that the hip joint is directly above the knee joint. Flexion‐extension occurs along the Z axis, abduction‐adduction along the *X* axis, and long‐axis rotation along the *Y* axis.

**Figure 2:**
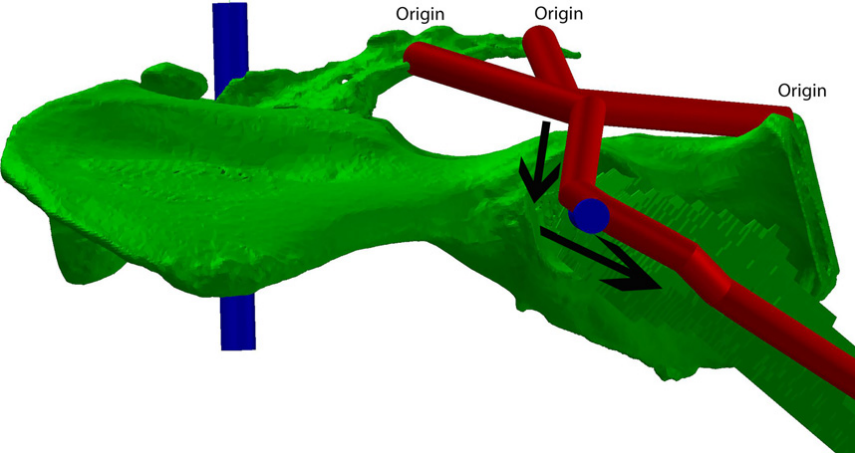
Gluteus maximus sites of origin and muscle paths (indicated by the black arrows). Note there are three origins chosen because of the muscle's strong attachments to the pelvis at these sites.

The 3D co‐ordinate information on bone and joint positions was then used as a basis for creation of a multi‐body dynamics model in gaitsym (Fig. 1). Detailed information on how joint centres and segment rotations were defined can be found in Supporting Information, but these also follow previous studies (Bates & Schachner, 2012; Bates et al. 2012a,b, 2015; Maidment et al. 2014). The gaitsym model included all the dissection information of each muscle (origin, insertion, fibre length, tendon length, PCSA). The deepest muscles were mapped on to the gaitsym model first, followed by those that were more superficial. We also used the skin outline of the gorilla extracted from the CT scan to constrain the maximum extent of the superficial muscles. In the case of fan‐shaped muscles (e.g. gluteals), multiple muscle tendon paths that converged onto a single line of action at their insertion were used (Fig. 2). This meant multiple origin sites could be defined where there were multiple distinct attachment sites. Equally, if the muscle was strongly attached to a large area, multiple origins across that area were modelled (Fig. 2). Each muscle path was checked as the joint was flexed and extended to ensure that the muscle did not pass through bones or other muscles. Additional ‘via points’ were added whenever necessary to guide muscle paths to prevent collisions and penetrations into other hard and soft tissue structures.

We manipulated our model to extract muscle moment arms one joint at a time, as in our previous studies (Bates & Schachner, 2012; Bates et al. 2012a,b, 2015; Maidment et al. 2014). Specifically, in a series of simulations, one joint was allowed to rotate through the maximum range of motion measured in kinematic studies of gorilla locomotion (Isler, 2005; DeSilva, 2008; Watson et al. 2009), while all other joints remained locked in their ‘neutral’ posture (Fig. 1). For example, data on hip muscles were extracted during rotation of the hip from 50° extension to 50° flexion while all distal limbs joints remained fixed at zero degrees, as in previous modelling (Murray et al. 1995; Pigeon et al. 1996; Brown et al. 2003; Hutchinson et al. 2005, 2014; Chan & Moran, 2006; Ogihara et al. 2009; Arnold et al. 2010; Bates & Schachner, 2012; Bates et al. 2012a,b, 2015; O'Neill et al. 2013; Maidment et al. 2014) and experimental studies (Young et al. 1993; Thorpe et al. 1999; Graham & Scott, 2003; Payne et al. 2006b; Smith et al. 2007; Channon et al. 2010; Michilsens et al. 2010; Holowka & O'Neill, 2013). Explanation of the joint co‐ordinate system used in relation to those of the experimental studies of non‐human ape kinematics (Isler, 2005; DeSilva, 2008; Watson et al. 2009) can be found in Supporting Information. It should be noted that the values taken from Watson et al. (2009) were measured manually from the graphs provided in their paper, whereas exact values were used from the studies of Isler (2005) and DeSilva (2008). In addition, a relatively qualitative method was used in DeSilva (2008) to obtain joint angles and this should be taken into account when interpreting the corresponding findings and conclusions related in this study.

Initially, we generated flexion‐extension moment arm data with all joints held at zero degrees abduction‐adduction and long‐axis rotation (i.e. in the neutral posture with respect to these axes). However, locomotion inherently involves 3D segment rotations, particularly at the hip and ankle in gorillas (Isler, 2005). Therefore, to provide the first insight into the effect of 3D limb position on muscle moments in the gorilla, and to extend our assessment of locomotor optimality into 3D, we also generated moment arms across a range of flexion‐extension angles with the hip abducted at 0°, 30° and 50°, and adducted at 20° (see Supporting Information). This range covers the majority of the kinematic ranges used during climbing, terrestrial quadrupedalism and bipedal walking (Isler, 2005; Watson et al. 2009) and is within *in vivo* baseline range of motion (Hammond, 2014). In the case of the ankle joint, our decision to output moment arms from a spectrum of postures was equally motivated by uncertainty in defining the most appropriate orientations of joint axes and (intrinsically linked to this uncertainty) the exact 3D rotations utilised by gorillas during locomotion. Preliminary studies have shown that some ankle abduction (up to 10°) occurs during climbing in western lowland gorillas (DeSilva, 2008), and thus flexion‐extension moment arms were generated across a range (0–110°) of flexion‐extension angles (DeSilva, 2008; Watson et al. 2009) when the ankle was abducted to 0°, 10° and 20°, thereby yielding values across a spectrum of 3D ankle postures. Our model file can be found in the Supporting Information material and is thus freely available to workers who wish to experiment with alternative joint axis orientations and motions. Raw moment arm data can also be found at https://doi.org/10.17638/datacat.liverpool.ac.uk/267 in the file ‘IndividualMuscleMomentArms.xlsx’.

#### Sensitivity analysis

To examine the effects of modifying origins/insertions on moment arms generated, we conducted sensitivity analyses of the gastrocnemius lateral and medial head around the knee, and gluteus minimus medial head 3 (the most middle part of our gluteus minumus medial head) and rectus femoris around the hip. The gastrocnemius and rectus femoris were chosen as the origins of both heads were extremely close to the knee and hip joints, respectively, and our previous work has shown high functional sensitivity in muscles with origins and/or insertions close to joint centres (Bates et al. 2012b). In gastrocnemius, the origins were moved superiorly by 0.01 m and in rectus femoris, the origin was moved superiorly by 0.005  and 0.01 m. O'Neill et al. (2013) conducted a sensitivity analysis on the gluteus minumus in their chimpanzee model. For direct comparison we shifted the gluteus minumus insertion superiorly and inferiorly by 0.01 m in our model.

### Muscle torque calculations

To examine how muscle torque varied with limb postures used during locomotion, we combined moment arms from our 3D model with muscle property data measured during dissection and from the literature (Diogo et al. 2010). We did not use the modelling software (which does have a number of different Hill‐type models with length and velocity dependent contraction) to produce torque estimates. Instead, all torque values provided herein were calculated under the assumption of maximum isometric muscle contraction according to:

(1) τ=PCSA×MA×FPUA

where τ is torque in Nm, PCSA is physiological cross‐sectional area in m^2^, MA is moment arm in metres and FPUA is the force per unit area (or maximum isometric stress) at maximum isometric contraction in Nm^−2^. Values between 200 000 and 400 000 Nm^−2^ (Pierrynowski, 1995; Zheng et al. 1998; Alexander, 2003; Umberger et al. 2003; Westneat, 2003) are widely reported for a range of species and muscles, and as such we used 300 000 Nm^−2^ because it is commonly used as an average value in modelling studies (Hutchinson, 2004; Bates et al. 2010; Bates & Falkingham, 2012; Sellers et al. 2013). Torque was calculated for the hip, knee and ankle joints, with all muscle parameters from dissected gorillas and from the literature (Diogo et al. 2010) adjusted to the size of the modelled gorilla under the assumption of geometric similarity (i.e. muscle masses scaled to body mass, and fibre lengths to body mass^0.33^; Alexander et al. 1981).

## Results

### Comparison of moment arms with Payne et al. (2006b)

**Figure 3:**
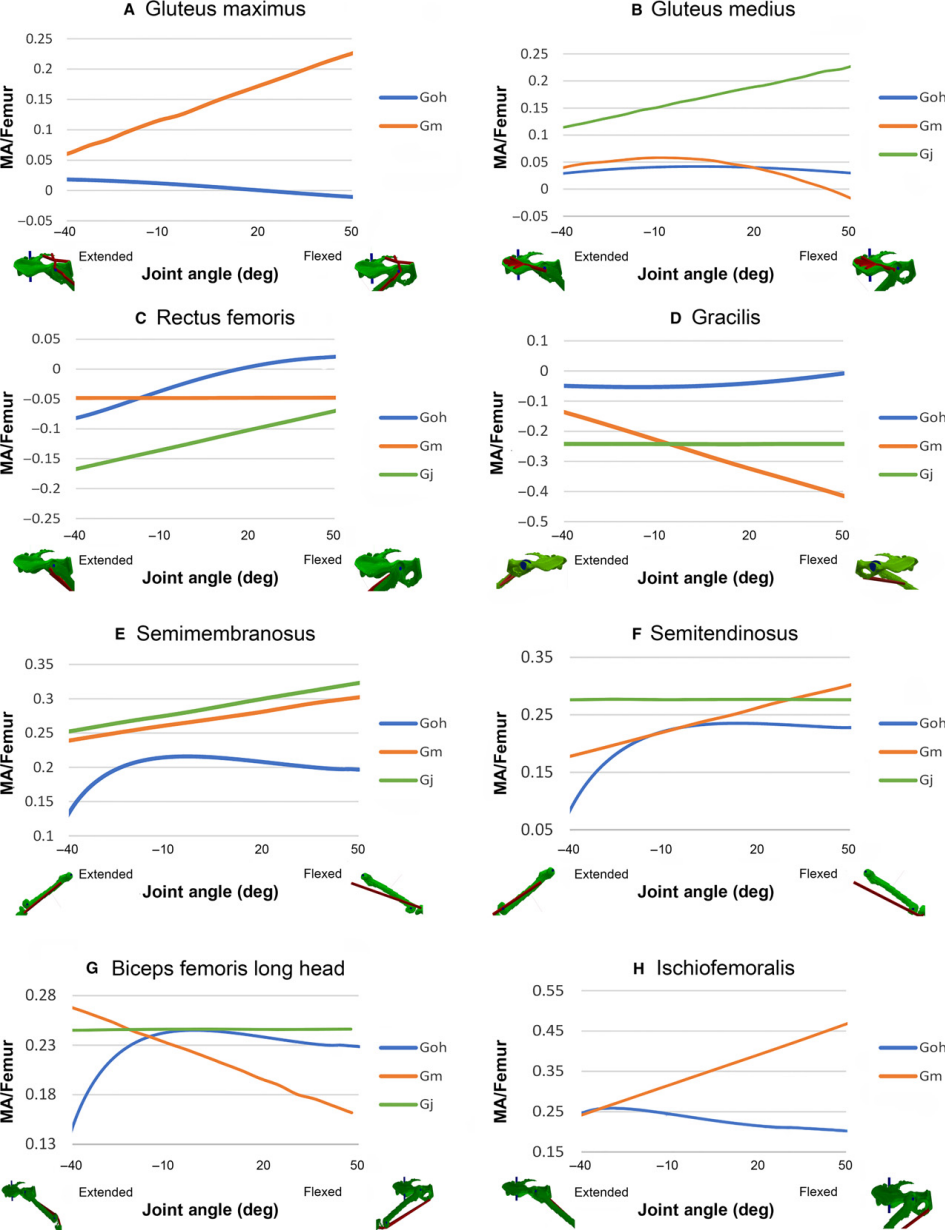
(A–F) Moment arms around hip for gluteus maximus, gluteus medius, rectus femoris, gracilis, semimembranosus, semitendinosus, biceps femoris long head and ischiofemoralis. Data from this study (Goh), eastern lowland gorilla (Gm) and western lowland gorlla (Gj) of Payne et al. (2006b). MA/femur refers to MA divided by femur length to account for differences in body size. *Y*‐axis: flexor moment is negative, extensor is positive. *X*‐axis: negative angle refers to extended, positive to flexed, and zero to the neutral position.

Moment arms for individual hip muscles from this study and that of Payne et al. (2006b) are shown in Fig. 3. The most striking difference was that although moment arm‐joint angle relationships from the current model were all non‐linear, all of the muscles reported in Payne et al. (2006b), with the exception of gluteus medius (for gorilla Gm), showed linear relationships or constant values across the joint angles tested (Fig. 3A,C–H). Substantial differences were also found for muscles with broad and irregularly‐shaped attachments. Gluteus maximus (Fig. 3A) and gluteus medius (Fig. 3B) from the model showed opposite trends and had magnitudes that were less than half of those in Payne et al. (2006b).

Muscles that did not cross directly above or below the hip joint also showed substantial differences. Our gracilis muscle (Fig. 3D) was predicted to be a much weaker flexor than that of Payne et al. (2006b; approximately four times less). Our biceps femoris long head (Fig. 3G) moment arm increased with increasing flexion, whereas the values given by Payne et al. (2006b) either decreased (Gm) or remained constant (Gj). Our ischiofemoralis moment arm decreased, whereas that of Payne et al. (2006b) increased as the hip was flexed (Fig. 3H). The moment arm values of rectus femoris in specimen Gj in Payne et al. (2006b) had a similar overall trend to that in our model, but differed in magnitude (Fig. 3C). Furthermore, the rectus femoris in our model changed predicted function from flexor to extensor as the hip was flexed, in contrast to Payne et al. (2006b), where it remained as a flexor.

**Figure 4:**
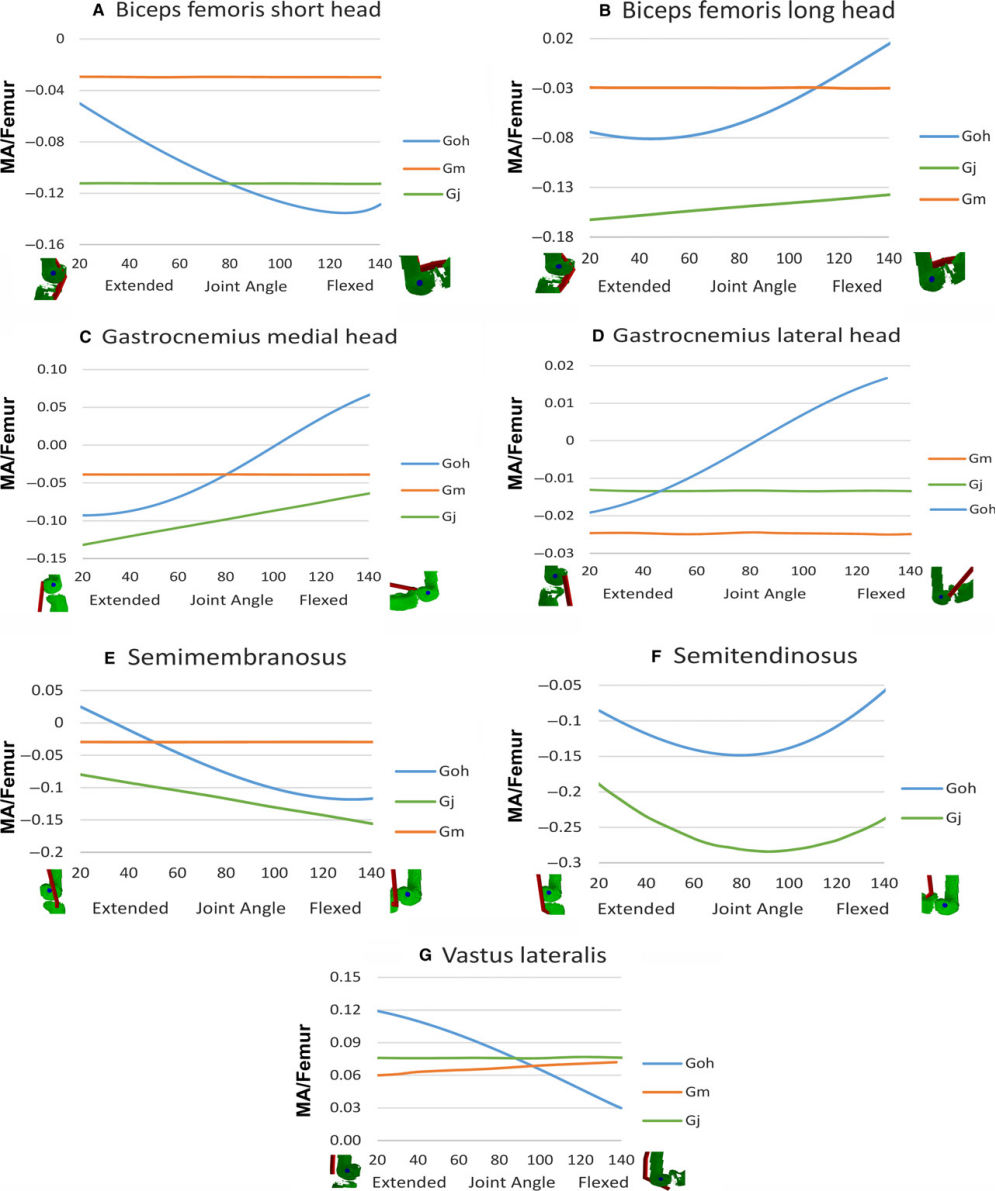
(A–G) Moment arms around knee for biceps femoris (short head), biceps femoris (long head), gastrocnemius (medial head), gastrocnemius (lateral head), semimembranosus, semitendinosus, and vastus lateralis. Data from this study (Goh), eastern lowland gorilla (Gm) and western lowland gorilla (Gj) of Payne et al. (2006b). MA/femur refers to MA divided by femur length to account for differences in body size. *Y*‐axis: flexor moment is negative, extensor is positive. *X*‐axis: zero is a fully extended knee, increasing values indicate increasing degrees of flexion.

Moment arms for individual knee muscles from this study and that of Payne et al. (2006b) are shown in Fig. 4. Only three muscles (biceps femoris short and long heads, and semimembranosus) showed similar overall values with the data of Payne et al. (2006b) (see Fig. 4A,B,E). Gastrocnemius medial and lateral heads, semitendinosus and vastus lateralis did not follow the data from Payne et al. (2006b) (Fig. 4C,D,F,G). However, values from the model change in curvilinear manner as opposed to the linear trends shown in most muscles of Payne et al. (2006b), with the exception of semitendinosus. The predicted function of the gastrocnemius lateral and medial heads, and semimembranosus changed between flexion and extension at extreme joint flexion in our model, but this did not occur in Payne et al. (2006b). Data for the vastus lateralis in Payne et al. (2006b) displayed an opposing trend to that of our model with respect to knee joint angle (Fig. 4G).

**Figure 5:**
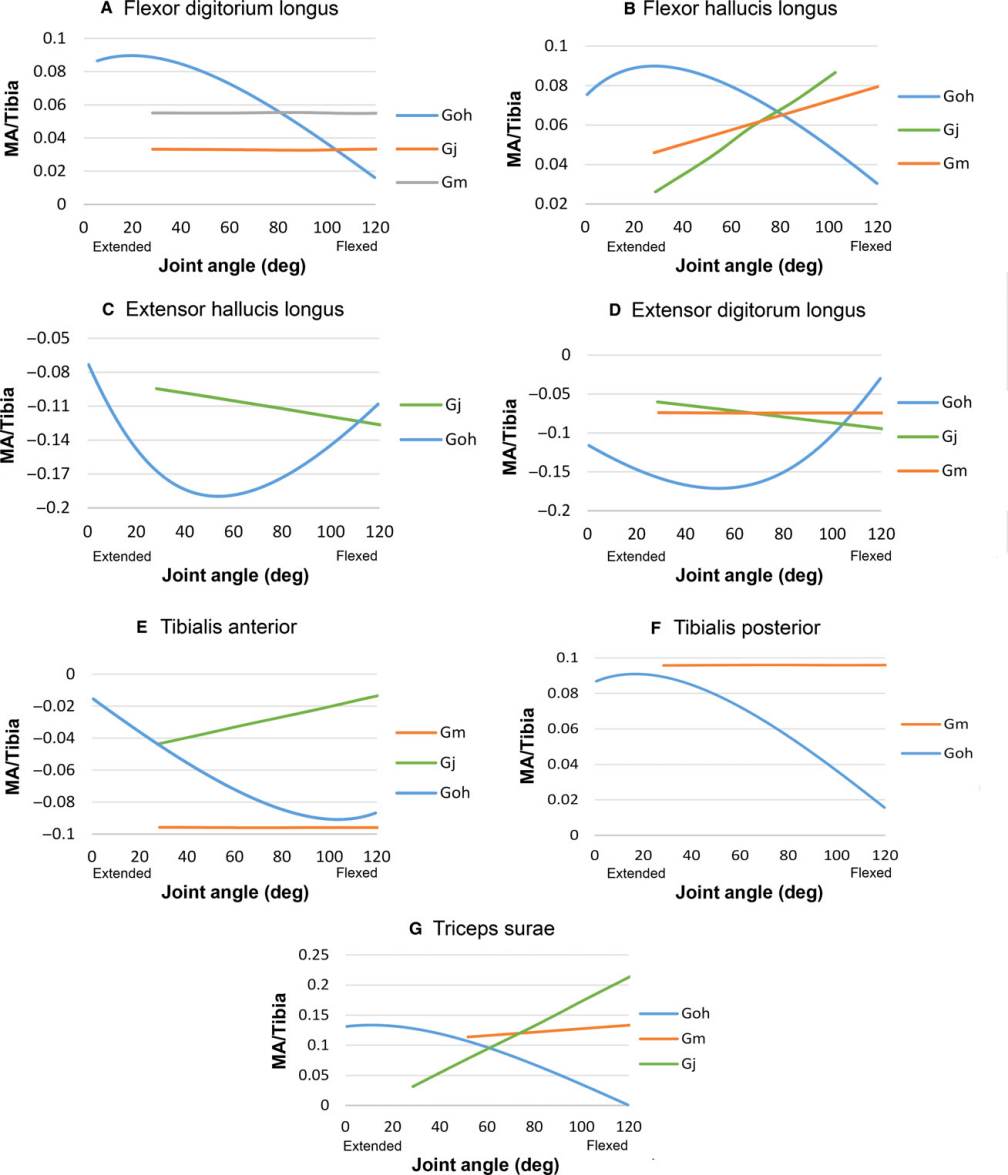
(A–G) Moment arms around ankle for flexor digitorum longus, flexor hallucis longus, extensor hallucis longus, extensor digitorum longus, tibialis anterior, tibialis posterior and triceps surae. Data from this study (Goh), eastern lowland gorilla (Gm) and western lowland gorlla (Gj) of Payne et al. (2006b). MA/tibia refers to MA divided by tibia length to account for differences in body size. *Y*‐axis: flexor moment is negative, extensor is positive. *X*‐axis: zero is a fully extended ankle, increasing values indicate increasing degrees of flexion.

Lastly, muscle moment arms around the ankle are reported in Fig. 5. Overall, larger magnitudes were found in the data generated by our model than those in Payne et al. (2006b). Further, there were differences in terms of direction of trend: our triceps surae, extensor hallucis longus and extensor digitorum longus moment arms decrease, but those of Payne et al. (2006b) increase with increasing dorsiflexion (Fig. 5C,D,G). Further, the moment arms from the model all show relatively more parabolic curves, whereas those of Payne et al. (2006b) either increase or decrease linearly with flexion‐extension of the joint, or remain constant (Fig. 5).

### Sensitivity analysis

**Figure 6:**
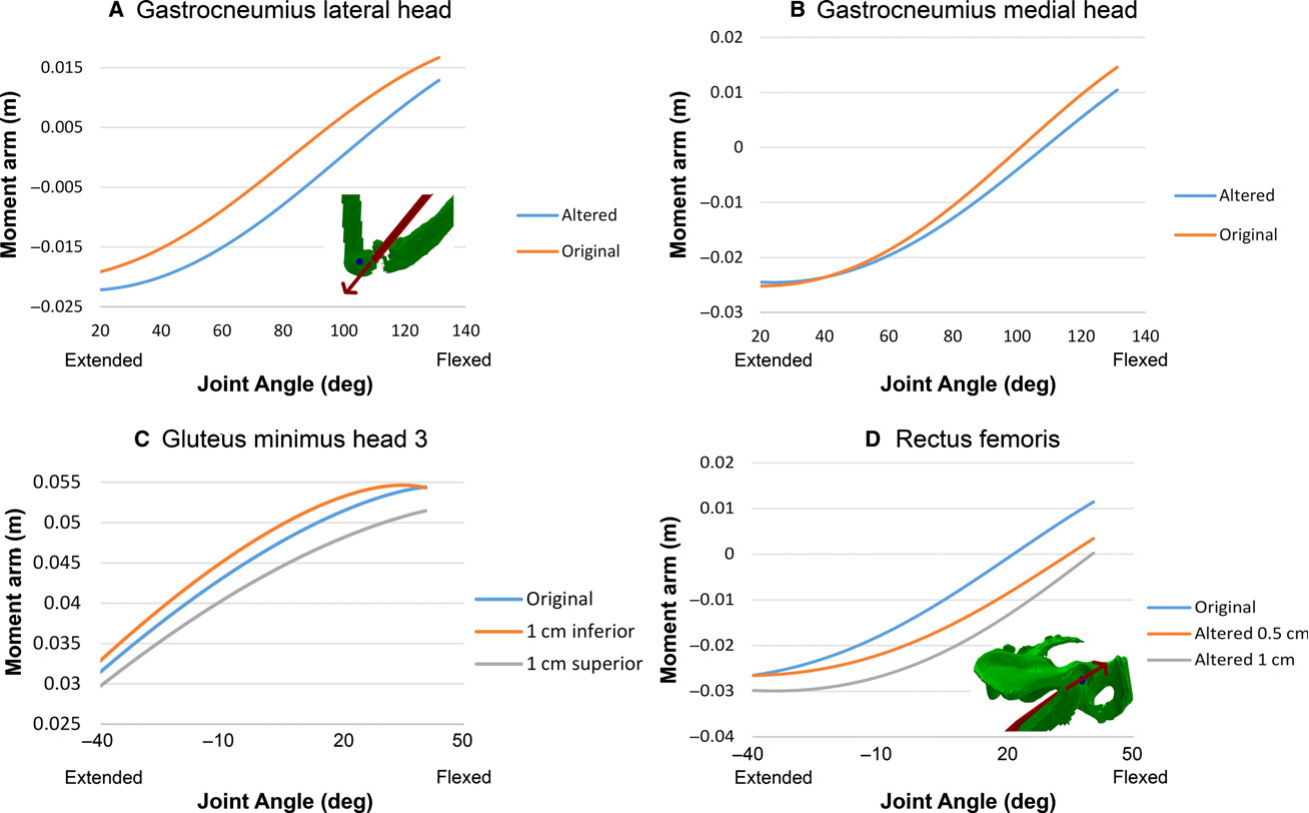
Sensitivity analysis for moment arms (in meters) gastrocnemius lateral and medial head around the knee (top), and gluteus minimus medial head 3 and rectus femoris around the hip joint (bottom). Pictures of gastrocnemius lateral head and rectus femoris have been included with muscles’ lines of action (red arrows) to portray shift in predicted function from flexor to extensor at extreme flexed positions.

Altering the position of muscle origins for the gastrocnemius lateral and medial heads, rectus femoris, and location of the insertion of gluteus minimus medial head produced relatively modest changes to moment arms (Fig. 6). All three muscles retained similar‐shaped curves. The gastrocnemius and rectus femoris muscles changed sign (signifying a predicted switch from flexor to extensor moment) at highly flexed postures (approximately 90° for gastrocnemius and 100° for rectus femoris in our initial model; Fig. 6). This can be explained in two ways. First, the close proximity of these muscle origins to the knee and hip joint, respectively, resulting in the muscle line of actions to cross inferior to the joints (e.g. see schematic drawing in Fig. 6), and hence the shift in predicted function to extension. Altering the origins caused the sign‐change to occur at slightly more flexed positions (> 100° for gastrocnemius and 120° for rectus femoris; Fig. 6). Secondly, the pelvis was orientated horizontally in our model, whereas other studies of apes have chosen to orientate it vertically, as in humans (e.g. O'Neill et al. 2013). With the hip orientated vertically, the origin of the rectus femoris lies above the hip joint and in this position it can only ever flex the hip. With the pelvis orientated horizontally, as in our model, the rectus femoris origin lies below the hip joint and it will inevitably change predicted function as the joint is rotated (Fig. 6). For gluteus minimus medial head 3, altering the insertion by 1 cm superiorly and inferiorly caused the moment arms generated to be slightly lower and higher, respectively (Fig. 6), reflecting the decreased/increased distance from the hip joint centre.

### Correlating moment arms and torque with joint angles utilised during different modes of locomotion

**Figure 7:**
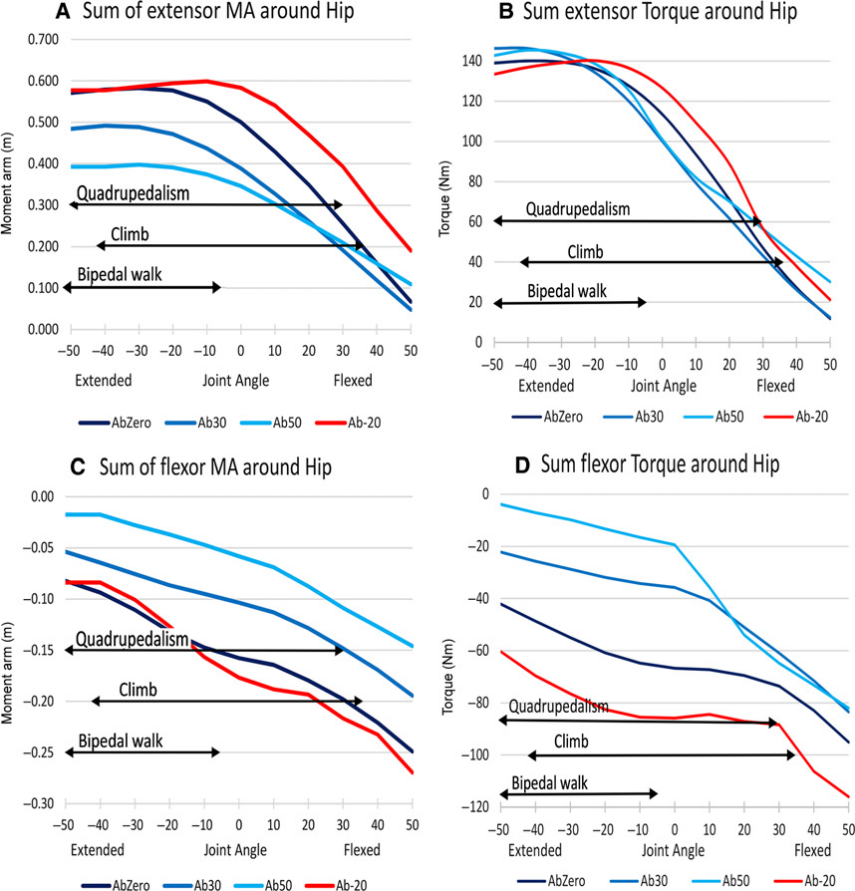
(A–D) Moment arm (in metres) and torque (Nm) at varying abduction angles around the hip. Flexed joint angles are positive, extended joint angles are negative, 0° refers to neutral position. AbZero refers to hip abducted at 0°, Ab30 at 30°, Ab50 at 50° and Ab‐20 adducted at 20°. Black arrows depict reported ranges of joint angles used for bipedal walking, quadrupedalism and climbing (Watson et al. 2009; Isler, 2005).

Data on the summed extensor and flexor moment arms and torque around the hip at several hip abduction angles are presented in Fig. 7. During climbing, maximum hip flexion occurs at maximum abduction, and maximum extension occurs at minimum abduction (Isler, 2005). Estimated values for bipedal walking and terrestrial quadrupedalism kinematics are taken from Watson et al. (2009). However, no abduction angles are available for gorilla bipedalism in the literature and thus data from chimpanzees were used as a proxy, where abduction occurs up to 30**°** (O'Neill et al. 2015). Overall, the summed extensor moment arms and torque decreased as the hip was flexed, and the flexor moment arms and torque decreased as the hip was extended (Fig. 7).

At extended postures (−50**°**), the summed extensor moment arm was higher by 45%, but torque lower by 3.4% at 0**°** abduction than at 50**°** abduction (Fig. 7A,B). This difference was most likely attributable to muscles medial to the hip joint. At 0**°** hip abduction, the summed extensor moment arm of the adductors (brevis, longus and magnus) was approximately 0.08 m greater than at 50**°** abduction. This difference was not observed with torque, as shown by the similar torque values at extended position (−50**°**). This was a result of the gluteus maximus having substantially higher torque (35 Nm), and gluteus medius changing from flexor at 0**°** abduction to extensor at 50**°** abduction, despite most of the other hip muscles having lower torque at 50**°** abduction than at 0**°** abduction. The summed flexor moment arm was relatively high (~ −0.08 m) at 0**°** hip abduction and 20**°** adduction than at the other abduction‐adduction postures tested (see Fig. 7C, ~ −0.05 m for 30**°** abduction; ~ −0.02 m for 50**°** abduction) at maximum flexion.

At flexed positions (50**°**), extensor torque at 50**°** hip abduction was 150% higher than at 0**°** abduction (Fig. 7). This was a result of gluteus medius having a high extensor torque (24 Nm) at 50**°** abduction, and acting as a flexor instead of an extensor at 0**°** hip abduction. Flexor moment and torque were consistently lower when the hip was abducted at 50**°** than 0**°**. This was attributable to muscles lateral to the hip joint. At 0**°** hip abduction, gluteus medius had a maximum flexor moment arm that was > 0.03 m greater, and a maximum torque 13 Nm greater, than at 50**°** abduction. Further, gluteus medius acted as a flexor at 0**°** abduction but changed to an extensor role at 50**°** abduction. It is interesting to note that flexor torques at 50**°** and 30**°** hip abduction were approximately equal at flexed positions (10**°**–50**°**) but flexor moment arms differed by 33%. This could be explained by gluteus maximus having a substantially higher flexor torque at flexed positions at 50**°** abduction (~ 13 Nm) than at 30° abduction (~ 3 Nm).

In the context of flexion‐extension kinematics for climbing, when the hip was abducted at 50° and at maximum flexion (50**°**), the extensor moment arm was relatively low and torque high (see Fig. 7A,B). Both flexor moment arm and torque were relatively low. At maximum extension (−50**°**), where minimal abduction occurs during climbing, extensor moment arms were relatively high and torque relatively similar to that of other hip abduction angles. Flexor moment arms and torque were both relatively high when hip was minimally abducted (Fig. 7C,D). Bipedal walking coincided with higher values of extensor moment arm at 0**°** and at 30**°** abduction compared with vertical climbing (see Fig. 7A). In contrast, bipedal walking range coincided with lower flexor moment arm and torque at 0**°** and 30**°** abduction (see Fig. 7C,D). Comparing the three locomotor modes of interest, the range of angles used during quadrupedalism coincided with higher moment arm and torque than that of climbing, but also encompassed joint angles where moment arm and torque were not at their highest. Bipedalism, however, used joint angle ranges that had higher extensor/flexor moment arms and torque (Fig. 7).

**Figure 8:**
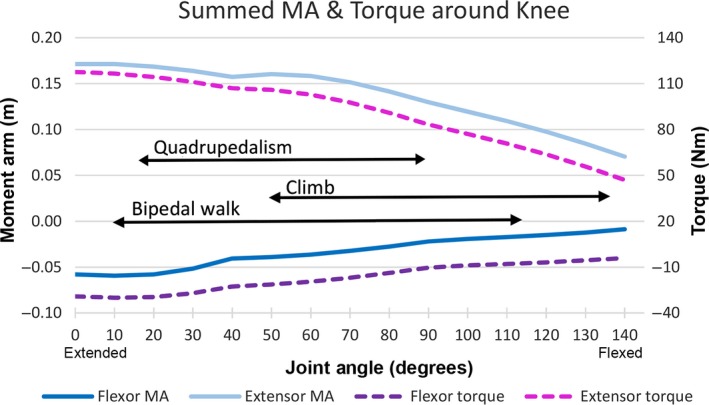
Moment arm (MA) (in metres) and torque (Nm) around the knee across a range of joint angles. 0° refers to extended position, 140° refers to flexed position. Black arrows depict reported ranges of joint angles used for bipedal walking, quadrupedalism and climbing (Watson et al. 2009).

Figure 8 shows data on the summed extensor and flexor moment arms and torques around the knee. Extensor moment arms and torque were consistently higher than flexor values (Fig. 8). Extensor and flexor moment arms and torque decreased as the knee flexed. For flexor moment arms, this was attributable to the gastrocnemius becoming an extensor at highly flexed postures of the knee, which is to some extent an artefact of our modelling approach and limitations on the constraints placed on muscle paths at extreme joint angles (see discussion above). Thus, climbing (which involves more flexed postures) corresponded with lower summed flexor and extensor moment arm and torque values than did terrestrial quadrupedalism and bipedal walking (Fig. 8), consistent with the trends observed in the majority of individual muscles (Fig. 4). Conversely, bipedal walking and quadrupedalism exclusively coincided with higher moment arm and torque values (Fig. 8).

**Figure 9:**
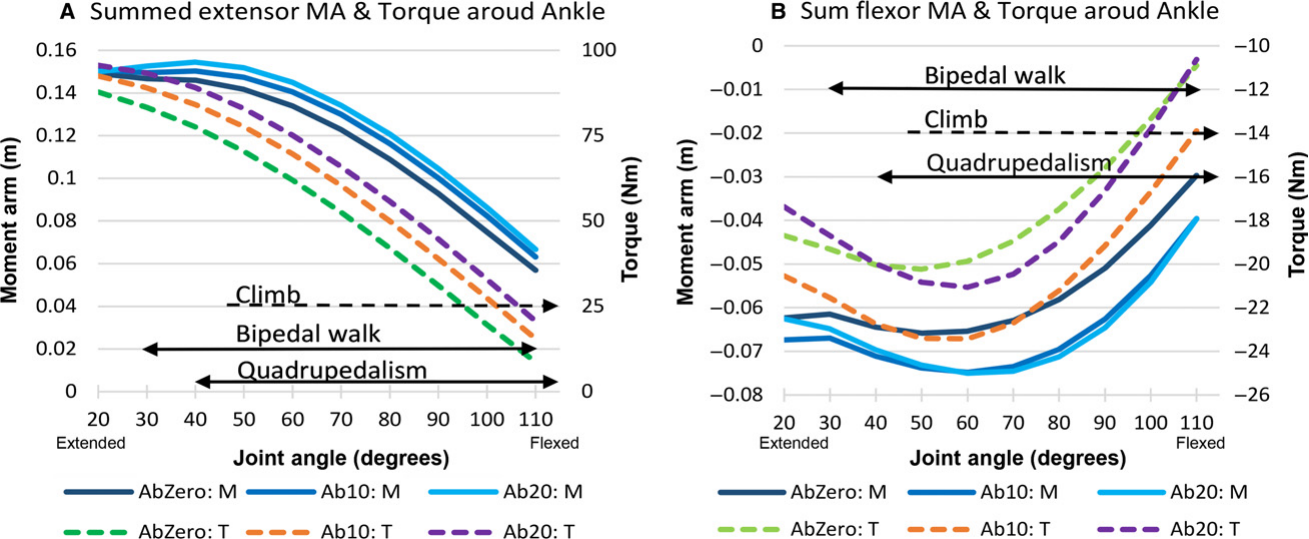
Sum extensor (A) and flexor (B) moment arm (MA – in metres) and torque (Nm) at different abduction angles around the ankle. Ankle is dorsiflexed as joint angle increases. T refers to torque; M to moment arm. AbZero refers to ankle abducted at 0°, Ab10 at 10°, Ab20 at 20°. Black arrows depict ranges of joint angles used for bipedal walking, quadrupedalism and climbing (Isler, 2005; Watson et al. 2009). In climbing, the ankle is abducted up to 10**°** and can be dorsiflexed by as much as 119**°** (DeSilva, 2008). Dotted black arrow used for climbing as only maximum dorsiflexion angle is known, unlike in bipedal walking where the exact range is known.

Data on the summed extensor and flexor moment arms and torques around the ankle at different abduction angles are shown in Fig. 9. Climbing and bipedal walking had similar maximum dorsiflexion angles. Bipedal walking coincided with higher values of extensor moment arm and torque but lower values of flexor moment arm and torque compared with quadrupedalism (Fig. 9). Summed extensor and flexor moment arms and torque decreased with dorsiflexion. The summed extensor moment arm peaked at ~ 40°, but torque peaked at a more extended position (~ 20°; Fig. 9A). Flexor moment arm and torque peaked at relatively similar positions (50° for torque and 60° for moment arm; Fig. 9B). Little difference in extensor moment arms and torque existed between 0**°** and 10**°** abduction (Fig. 9A). For summed extensor moment arm and torque, values at 0° abduction were the lowest, followed by those at 10° and 20° abduction (Fig. 9A). For summed flexor moment arm, from 40° to 100°, values at 0° abduction were the lowest, followed by values at 10° and 20° abduction, which were similar (Fig. 9B). On the other hand, summed flexor torque was lowest at 0° abduction, followed by at 20°, then 10° (Fig. 9B). This was a result of tibialis anterior having a higher torque (2.8 Nm) at 10° abduction than at 20°. Extended joint angles in bipedal walking (30**°**–40**°**) corresponded with the high extensor moment arm (Fig. 9A). This corresponded also with high flexor moment arm and torque, as flexor moment arm and torque increased as dorsiflexion increased from 40° to 60° and then decreased as dorsiflexion increased from 60° to 110° (Fig. 9B).

## Discussion

### Model‐based moment arms vs. previous experimental measures in Gorilla

Substantial differences were found between our model estimates and the moment arm data of Payne et al. (2006b), derived using a version of the experimental tendon travel method. First, moment arms predicted by our model were rarely either straight lines or constants, as was the case for the majority of those in Payne et al. (2006b), but instead were all curvilinear (Figs 5–7). Highly curvilinear trends for moment arm vs. joint angle curves are found wherever the methodological approach (whether computational or experimental) incorporates broadly realistic constraints on 3D muscle paths, as seen in earlier computational (Pigeon et al. 1996; Delp et al. 1999; Hutchinson et al. 2005; Ogihara et al. 2009; Bates et al. 2012b; O'Neill et al. 2013; Hutchinson et al. 2014; Maidment et al. 2014) and experimental studies (Young et al. 1992; Graham & Scott, 2003; Ackland et al. 2008; Michilsens et al. 2010). Some of these discrepancies can be explained by the differences in method used to collect previous moment arm data for gorilla; the specific tendon travel method approach used by Payne et al. (2006b) frequently yields linear relationships for a range of muscles in a variety of taxa (Thorpe et al. 1999; Smith et al. 2007; Channon et al. 2010). This is a product of the fact that in this version of the method, muscles are treated as a straight line, without any additional soft tissue or osteological constraints on the 3D path between origin and insertion. In our computer model, we were able to account for such constraints on muscle paths to a degree through the use of via points and wrapping surfaces. These constraints may also contribute to the large differences in magnitude found in some muscles; all hip muscles except biceps femoris long head (Fig. 3), knee: semitendinosus and vastus lateralis (Fig. 4F,G), ankle: flexor digitorum longus, extensor hallucis longus and extensor digitorum longus and tibialis posterior (Fig. 5A,C,D,F).

Muscle shape also appears to have contributed to differences in our results relative to those of Payne et al. (2006b). The gluteus maximus and gluteus medius (Fig. 3A,B) are both wide and irregular‐shaped, and they are therefore inherently difficult to represent accurately using a single straight line, as in Payne et al. (2006b). In our 3D model we were able to represent distinct regions with their own muscle path, with customised non‐linear behaviour specified by via points or wrapping surfaces. Related to this, differences between our data and those of Payne et al. (2006b) were more modest for long and thin muscles. For example, broadly similar magnitudes are recovered for biceps femoris long head at the hip (Fig. 3G), biceps femoris long (with gorilla Gm) and short (with gorilla Gj) heads, gastrocnemius medial and lateral heads (at extended positions) and semimembranosus at the knee (Fig. 4A‐E) and tibialis anterior, FHL and triceps surae at the ankle (Fig. 5B,E,G). Equally, around the hip, our rectus femoris showed similar trend direction to Gj, and extended positions in our semimembranosus and semitendinosus had similar trend directions with Gj and Gm, respectively, from Payne et al. (2006b); Fig. 3C,E,F).

Nonetheless, our model also produced a small number of unexpected switches in predicted muscle function, though these were restricted to extreme and often unrealistic limb postures. For example, we found changes in predicted function for gastrocnemius lateral and medial heads at the knee (Fig. 4C,D) as the joint reached highly flexed postures. This results from a combination of limitations in the software and the combined hip and knee postures used in this instance. Options for constraining muscle paths are restricted to either cylindrical wrapping surfaces or via points. Other MDA modelling packages (e.g. opensim) allow wrapping surfaces of varied geometry to be used in combination with via points to reproduce more complex constraints on muscle paths. Without such additional constraints, the gastrocnemius muscles in our model switch from flexors to extensors at highly flexed positions while the femur is held vertically in the standardised posture used herein. It is unlikely that this represents a biologically realistic posture, and it is likely that the hip would be much more flexed while the knee was at highly flexed postures (Isler, 2005).

Payne et al. (2006b) suggest that increased moment arms at flexed positions found in gluteus maximus, gluteus medius, gracilis, semimembranosus and semitendinosus around the hip (see Fig. 3A,B,D‐F) are an adaptation to vertical climbing and arboreal quadrupedalism, as these locomotor modes require the maintenance of flexed postures. However, our results contradict this conclusion, as the moment arms of gluteus maximus, gluteus medius and gracilis in our model did not increase in flexed postures (see Fig. 3A,B,D). Even after altering the origins of gluteus minimus, as shown in the sensitivity analysis, the trend remained the same (Fig. 6). As stated previously, to our knowledge, no other study of muscle moment arms in terrestrial tetrapods has found whole‐scale stabilisation or increases in extensor (anti‐gravity) muscle moments and torques in flexed limb postures. The tendency for the moment arms of hip extensors such as gluteus maximus, gluteus medius, semimembranosus and semitendinosus to decrease with increasing flexion appears to be a fundamental geometric constraint, as these muscles will tend to be pulled towards the joint as the hip is flexed, thus decreasing the distance from the muscles’ lines of action to the joint centre (see Fig. 3A,B,E,F). This pattern has also been observed by O'Neill et al. (2013) and in human studies (Hoy et al. 1990; Visser et al. 1990), where hip extensors such as gluteus maximus proprius (gluteus maximus), semimembranosus and semitendinosus decreased in moment arms as the hip was flexed. Further, the vastus lateralis in Payne et al. (2006b) had an increasing moment arm with increasing knee flexion. This is theoretically impossible unless there is a bony protrusion/soft tissue that pushes the muscle away as the knee is flexed. In our model and other studies (Visser et al. 1990; Spoor & Van Leeuwen, 1992; Krevolin et al. 2004), similar knee extensors are pulled towards the joint as the knee is flexed, causing moment arm to decrease with increasing flexion. In the absence of a clear anatomical mechanism responsible for maintaining or increasing extensor moment arms at flexed postures, we suggest that our model, with its increased anatomical detail, provides more accurate qualitative and quantitative representations of muscle moment arms in the gorilla. With this in mind, we now visit the issue of limb muscle moment arms and torques in the context of adaptations for different locomotor modes, specifically vertical climbing, terrestrial quadrupedalism and bipedalism.

### Moment arms and torques during different modes of locomotion

Around the hip, although the summed extensor moment arm when abducted at 50**°** was relatively low at maximum flexion (50**°**) as compared with when the hip was adducted at 20**°**, the torque was high (Fig. 7A,B). Explanations for this include the large gluteus medius being a good extensor at 50**°** and the presence of large muscles (i.e. gluteus medius) that could generate power and facilitate pushing the body upward during climbing. At maximum extension, extensor moment arms and torque were relatively high at 0° hip abduction. This is expected, as the hip extensors would be active to maintain extended postures at the end of the support phase of climbing.

The summed flexor moment arms (and torque) at 0**°** hip abduction and maximum extension were relatively high (~ −0.08 m) compared with the more abducted joint angles (~ −0.06 m when hip is abducted at 30**°**; ~ −0.02 m when hip is abducted at 50**°**; Fig. 7C). This would enable the gorilla to flex its hip more efficiently and powerfully from an extended position while also keeping its body close to the support during climbing (provided the knees are flexed simultaneously with the hip). It has been shown that all great apes, including humans, keep their bodies close to the substrate during climbing, as it is safer and more energetically efficient to keep the body centre of mass closer to the support during vertical climbing (Cartmill & Milton, 1977; DeSilva, 2008; Venkataraman et al. 2013). The decrease in the moment arm between the body centre of mass and the support reduces the torque and subsequently the muscle forces required to counteract downward force resulting in toppling (Cartmill & Milton, 1977; DeSilva, 2008; Venkataraman et al. 2013).

Joint angles used during bipedal walking, and in most cases quadrupedalism, coincided with relatively high values for extensor moment arms and torque than climbing (Fig. 7A,B). Bipedal walking in particular involves more extended angles (Fig. 7) than flexed angles throughout the limb, although it should be noted that the existing data for bipedal walking kinematics in gorillas is sparse (Watson et al. 2009). Our results indicate that the geometric arrangement of hip extensors in the gorilla are more mechanically effective for bipedal walking (and to a great extent terrestrial quadrupedalism), contradicting the suggestion of Payne et al. (2006b) of an adaptation to maintain high moment arms at flexed postures around the hip in both terrestrial and arboreal contexts (see above). Our extensor moment arm (and torque) peaked at extended postures (−20° to −40°; Fig. 7A), and not at flexed postures as suggested by Payne et al. (2006b). Additionally, the adductors were important extensors at 0**°**–30**°** abduction. Hence this would likely assist in extension during terrestrial bipedal walking, as chimpanzees abduct their hips to 14**°**–30**°** (O'Neill et al. 2015) during terrestrial bipedal walking.

Only in hip flexors did the climbing joint angle range coincide with higher moment arms and torque than that of bipedalism and quadrupedalism (Fig. 7). As regards extensors, our data suggest that gorillas have the ability to propel the body powerfully upwards in the last phase of hind limb contact with a vertical substrate. This appears to be a result of geometric constraints on limb moment arms, with extensor muscle being drawn closer to joint centres as limb segments become increasingly flexed (Brown et al. 2003; Bates & Schachner, 2012; Bates et al. 2012a,b, 2015; O'Neill et al. 2013; Hutchinson et al. 2014; Maidment et al. 2014). Thus moment arms and torques are relatively lower across the more flexed postures utilised in vertical climbing compared with quadrupedalism and particularly bipedal walking (Fig. 7).

Extensor muscle moment arm and torque were consistently higher than flexor moment arm and torque at the knee (Fig. 8). Zihlman et al. (2011) have shown that gorillas have larger knee extensors than flexors, for propulsion and stability, lending more evidence to the importance of knee extension in gorilla locomotion. Extensor moment arm and torque decreased as the knee was flexed and as a result, bipedal and quadrupedal walking coincided with higher values of moment arm and torque than climbing.

Ankle extensor and flexor muscle moment arm and torque values decreased with increasing dorsiflexion (Fig. 9A,B). As with more proximal joints, this is expected, as the muscles that dorsiflex the foot will be pushed closer to the joint centre, and the muscles that plantarflex the foot will be flattened against the joint with increasing dorsiflexion. Our findings suggest that ankle abduction during climbing does not compromise the efficiency or power of the extensors, and in fact increases moment arm and torque in the ankle flexors. For extensor muscle moment arms and torque, there were minimal differences between the ankle abducted at 10**°** or at 0**°** (Fig. 9A). For flexor moment arm and torque, values were higher at 10**°** or 20**°** abduction than at 0**°** (Fig. 9B). The peak extensor moment arm occurred at a relatively extended posture (40°, see Fig. 9A), and the peak flexor moment arm and torque at a less extended posture (60°, see Fig. 9B). This enables the extensors of the stance leg to effectively extend the ankle during bipedal walking (just before maximum extension at 30**°**) to propel the leg forward, and the flexors (at 60°) to be effective in dorsiflexing the foot during swing phase. On present evidence, bipedal walking involves slightly more extended joint angles at the ankle than do quadrupedalism and climbing, and hence our results may suggest that the former is characterised by more mechanically optimal kinematics (Fig. 9). However, the relatively small kinematic datasets, and their estimation of joint angles from single‐plane external video (Watson et al. 2009), means that relatively small differences between ankle joint ranges of these locomotor modes should be viewed with caution.

## Conclusion

In this study substantial differences were found in moment arm trends and magnitudes between our model and previous experimental tendon travel data (Payne et al. 2006b). Much of the noted disparity can be attributed largely to methodological differences between the two studies, stressing the importance of accounting for complexities in muscle paths/shapes when collecting moment arm data. Our results also contradict and cast significant doubt upon the suggestion by Payne et al. (2006b) that higher moment arms at flexed positions in hip extensors primarily might be an adaptation to vertical climbing and arboreal quadrupedalism, as these locomotor modes require the maintenance of flexed postures. We found that the reported relatively extended hip joint angles during bipedal walking, and to a great extent quadrupedalism, coincided mostly with higher moment arms and torques around the hip, knee and ankle, with lower moment arms and torques found at the more flexed angles typically used in vertical climbing. This indicates that the ability of a gorilla to walk bipedally is not restricted by musculoskeletal adaptations for vertical climbing and quadrupedalism, at least in terms of moment arms and torques about those joints. Hence bipedal kinematics and gross muscle mechanics may explain why bipedalism is used especially to negotiate small and oddly angled arboreal supports to obtain food (Stanford, 2006; Thorpe et al. 2007b). This perhaps provides some evidence in line with the argument of Myatt et al. (2011) and Neufuss et al. (2014) that the retention of locomotor plasticity may have been selected for in gorillas. However, more interspecies comparisons of moment arm data need to be carried out to test this hypothesis.

## Author contributions

Colleen Goh carried out the dissection, built the model, did data analysis and drafted the manuscript. Mary Blanchard assisted in the dissection and data analysis. Robin Crompton assisted with analysis of the results and critically reviewed the manuscript. Michael Gunther provided invaluable help with dissection, especially for the foot muscles. Sophie Macaulay provided comments on the manuscript and assisted with dissection. Karl Bates helped with building the model, data analysis, critical manuscript review and approval. The authors have no conflict of interest to declare.

## Supporting information


**Table S1.** Muscle mass, fascicle length (FL) and physiological cross‐sectional area (PCSA) of muscles. Mass, FL and PCSA were scaled to body mass of the gorilla from CT scan, as shown in methods.
**Fig. S1.** Conventions for joint angle measurements used in the previous studies of *Gorilla* muscle moment arms (Payne et al. 2006b) and limb kinematics (Isler, 2005; Watson et al. 2009) compared with those used in our model. Values derived for the posture shown above using the convention used in our model are indicated by the green curves and blue angles/text, whereas those of previous studies are represented by the black curves and angles/text. The dashed green lines indicate a joint angle of zero (neither flexed nor extended) for each segment in the convention used in our model. All joint angle values and ranges from past studies were converted to the convention used in our model and shown above for the purpose of the comparisons made in the main text.
**Fig. S2.** (A) Hip, (B) knee and (C) ankle in flexion. Black arrows show direction of flexion.

**Appendix S1.** Additional information on Material and methods.
**Appendix S2.** Individualmusclemomentarms.
**Appendix S3.** GorillaHindlimbModel.xml: Gorilla hind limb musculoskeletal model as human readable xml file, suitable for gaitsym.

**Video S1.** Hip animation: flexion and extension of hip.

**Video S2.** Knee animation: flexion and extension of knee.

**Video S3.** Ankle animation: flexion and extension of ankle.
