# Supplementary material for: A 3D musculoskeletal model of the western lowland gorilla hind limb: moment arms and torque of the hip, knee and ankle
Source: J Anat. 2017 Jul 17;231(4):568–84. doi: 10.1111/joa.12651 (PMC5603783; doi:10.1111/joa.12651)
Supplement: Supplementary file 1 — Table S1. Muscle mass, fascicle length (FL) and physiological cross‐sectional area (PCSA) of muscles. Mass, FL and PCSA were scaled to body mass of the gorilla from CT scan, as shown in methods. Fig. S1. Conventions for joint angle measurements used in the previous studies of Gorilla muscle moment arms (Payne et al. 2006b) and limb kinematics (Isler, 2005; Watson et al. 2009) compared with those used in our model. Values derived for the posture shown above using the convention used in our model are indicated by the green curves and blue angles/text, whereas those of previous studies are represented by the black curves and angles/text. The dashed green lines indicate a joint angle of zero (neither flexed nor extended) for each segment in the convention used in our model. All joint angle values and ranges from past studies were converted to the convention used in our model and shown above for the purpose of the comparisons made in the main text. Fig. S2. (A) Hip, (B) knee and (C) ankle in flexion. Black arrows show direction of flexion. [file JOA-231-568-s001.docx]

**Electronic Supplementary Material 1 (ESM1) for**

**A 3D musculoskeletal model of the western lowland gorilla hind limb: moment arms and torque of the hip, knee and ankle**

Colleen Goh, Mary L. Blanchard, Robin H. Crompton, Michael M. Gunther, Sophie Macaulay and Karl T. Bates

**Additional information on materials and methods**

**Subject data**

Ideally, anatomical dissection and 3D computer model construction would be performed on the same specimen. However, a single gorilla specimen available to us for this study was not suitable for modelling due to skeletal damage and partial dissection carried out by other researcher. Therefore, we used qualitative information collected during dissection of the damaged specimen [supported by reference to [Diogo et al. (2010](#_ENREF_24))] to map muscle origins, insertions and 3D paths on to the model constructed from a CT scan of another gorilla. Quantitative data on muscle properties were also collected during dissection and merged with the previously published data ([Payne et al. 2006b](#_ENREF_49); [Payne et al. 2006a](#_ENREF_48)) in our model (see further details below). To our knowledge, the magnitude of inaccuracy in defining 3D musculoskeletal geometry and muscle performance predictions resulting from mixing data from different individuals within a species has never been quantified (although see [Carbone et al. (2012](#_ENREF_15)) for an analysis of similar errors on functional predictions). Recently, [Hutchinson et al. (2014](#_ENREF_35)) compared a highly detailed, dissection-based 3D model of a single ostrich to an earlier model constructed from a museum skeleton without associated dissection data ([Bates and Schachner 2012](#_ENREF_7)), and found general quantitative agreement and few (if any) qualitative differences in muscle moment arms. Human studies ([Rehbinder and Martin 2001](#_ENREF_53); [Sellers et al. 2003](#_ENREF_59); [Sellers et al. 2004](#_ENREF_60); [Wang et al. 2004](#_ENREF_73); [Pennestri et al. 2007](#_ENREF_50)) and other nonhuman primate studies ([Sellers et al. 2010](#_ENREF_62); [O'Neill et al. 2013](#_ENREF_46); [Sellers et al. 2013](#_ENREF_58)) also routinely construct models on the basis of muscle locations and paths that are considered typical or characteristic of the species in question, mixed with muscle size and architecture data available in the literature (see [Carbone et al. (2015](#_ENREF_14)) for an example of an exception). Therefore, we feel that for the purposes of producing a generic species-level 3D model of the western lowland gorilla to evaluate the findings of a previous study ([Payne et al. 2006b](#_ENREF_49)), our model is adequate.

Table S1. Muscle mass, fascicle length (FL) and physiological cross-sectional area (PCSA) of muscles. Mass, FL and PCSA were scaled to body mass of the gorilla from CT scan, as shown in methods.

| **Muscle name** | **Mass (g)** | **Mass scaled (g)** | **Length (mm)** | **FL** | **FL scaled** | **PCSA** | **PCSA scaled** |
| --- | --- | --- | --- | --- | --- | --- | --- |
| **Adductor brevis** | 85.7 | 179.9 | 180 | 107 | 137 | 0.8 | 2.3 |
| **Abductor hallucis** | 20.8 | 43.8 | 128 | 68 | 87 | 0.3 | 0.5 |
| **Adductor longus** | 78 | 163.8 | 200 | 156 | 200 | 0.5 | 0.8 |
| **Adductor magnus lateralis** | 224.6 | 471.7 | 320 | 270 | 346 | 0.8 | 1.3 |
| **Adductor magnus medialis** | 133.3 | 279.8 | 260 | 239 | 306 | 0.5 | 0.9 |
| **Biceps femoris long head** | 111.4 | 233.9 | 280 | 169 | 216 | 0.6 | 1 |
| **Biceps femoris short head** | 41.3 | 86.7 | 180 | 123 | 157 | 0.3 | 0.5 |
| **Extensor digitorum brevis 2** | 1.8 | 3.7 | 97 | 65 | 83 | 0 | 0 |
| **Extensor digitorum longus** | 30.8 | 64.7 | 155 | 135 | 173 | 0.2 | 0.4 |
| **Extensor hallucis longus** | 20.8 | 43.7 | 225 | 117 | 150 | 0.2 | 0.3 |
| **Fibularis brevis** | 23.7 | 49.8 | 200 | 70 | 90 | 0.3 | 0.5 |
| **Fibularis longus** | 39.9 | 83.9 | 232 | 49 | 63 | 0.8 | 1.3 |
| **Flexor digitorum longus** | 41.3 | 86.6 | 333 | 65 | 83 | 0.6 | 1 |
| **Flexor hallucis longus** | 19 | 39.8 | 270 | 88 | 113 | 0.2 | 0.3 |
| **Gastrocnemius lateralis** | 35.4 | 74.2 | 255 | 91 | 117 | 0.4 | 0.6 |
| **Gastrocnemius medialis** | 72 | 151.1 | 230 | 69 | 89 | 1 | 1.6 |
| **Gemellus inferior** | 5.4 | 11.4 | 115 | 55 | 70 | 0.1 | 0.2 |
| **Gluteus maximus ischiofemoralis** | 356.9 | 749.6 | 230 | 150 | 192 | 2.2 | 3.7 |
| **Gluteus maximus propius** | 127.8 | 268.3 | 180 | 126 | 161 | 1 | 1.6 |
| **Gluteus medius** | 531.6 | 1116.4 | 263 | 154 | 197 | 3.3 | 5.3 |
| **Gluteus minimus lateralis** | 49.8 | 104.7 | 180 | 100 | 128 | 0.5 | 0.8 |
| **Gluteus minimus medialis** | 52.9 | 111.1 | 111 | 55 | 70 | 0.9 | 1.5 |
| **Gracilis** | 120.6 | 253.3 | 310 | 284 | 364 | 0.4 | 0.7 |
| **Iliacus** | 110.4 | 231.9 | - | 220 | 282 | 0.5 | 0.8 |
| **Iliocapsularis** | 6.5 | 13.6 | 98 | 72 | 92 | 0.1 | 0.1 |
| **Ischiocondylica** | 177.3 | 372.4 | 395 | 328 | 420 | 0.5 | 0.8 |
| **Obturator externus inferior head** | 58.1 | 121.9 | 110 | 57 | 72 | 1 | 1.6 |
| **Obturator externus superior head** | 5.1 | 10.6 | 111 | 108 | 138 | 0 | 0.1 |
| **Obturator internus** | 6.9 | 14.4 | 58 | 30 | 38 | 0.2 | 0.4 |
| **Pectineus** | 28.9 | 60.7 | 136 | 127 | 163 | 0.2 | 0.4 |
| **Popliteus** | 21.9 | 46.1 | 132 | 27 | 35 | 0.8 | 1.2 |
| **Psoas major** | 55.2 | 116 | 240 | 240 | 307 | 0.2 | 0.4 |
| **Quadratus femoris** | 27.1 | 56.9 | 105 | 82 | 105 | 0.3 | 0.5 |
| **Rectus femoris** | 95.2 | 199.9 | 295 | 88 | 113 | 1 | 1.7 |
| **Sartorius** | 59 | 123.9 | 285 | 280 | 358 | 0.2 | 0.3 |
| **Semi-membranosus** | 54.8 | 115.2 | 270 | 227 | 291 | 0.2 | 0.4 |
| **Semitendinosus** | 172.7 | 362.8 | 350 | 348 | 446 | 0.5 | 0.8 |
| **Soleus** | 158.8 | 333.6 | 31.3 | 46 | 58 | 3.3 | 5.4 |
| **Tibialis anterior** | 155.1 | 325.6 | 254 | 48 | 61 | 1.5 | 5 |
| **Tibialis posterior** | 49.8 | 104.7 | 242 | 44 | 56 | 1.1 | 1.8 |
| **Vastus intermedius** | 15.1 | 31.6 | 190 | 110 | 141 | 0.1 | 0.2 |
| **Vastus lateralis** | 237.2 | 498.2 | 275 | 123 | 157 | 1.8 | 3 |
| **Vastus medialis** | 349.5 | 734 | 280 | 86 | 110 | 3.8 | 6.3 |

**Joint centres and segment rotations**

Joint centre positions were defined qualitatively by fitting spheres to each joint, with the centre of each sphere taken as the joint centre of rotation ([Hutchinson et al. 2005](#_ENREF_34); [Bates and Schachner 2012](#_ENREF_7); [Bates et al. 2012a](#_ENREF_8); [Bates et al. 2012b](#_ENREF_10); [Maidment et al. 2014](#_ENREF_39)). Each initial joint centre position was checked by flexing and extending the joints around the estimated joint centre in Maya to ensure that an appropriate range of motion was possible at each joint. The bones were then rotated about these joint centres into a ‘neutral position’ (Figure 1). The neutral position chosen herein refers to joint angles being 0**°**±0.001 for hip, knee and ankle for all three rotational axes. Figure 1 shows the neutral position and how the hip, knee and ankle joints, are directly above each other (i.e. in the same y plane and z plane). The model was posed so that hip flexion-extension axis was horizontal and parallel to the global z-axis, the adduction-abduction axis was horizontal and aligned to the global x-axis, and the femoral long-axis rotation axis vertical and parallel to the global y-axis in the initial neutral posture (refer to Figure 1 for neutral position and axes orientations). During motion, or when posture is statically altered, our joint axes rotate following the scheme outlined in [Wu et al. (2002](#_ENREF_77)). Specifically, the flexion-extension axes were fixed to the joint’s proximal segment, while long axis rotation axes were fixed to the joint’s distal body. Abduction-adduction axes move accordingly to remain orthogonal to the flexion-extension and long axis rotation axes depending on the rotations involved. Specifically, the abduction-adduction axis is rotated by rotation about the flexion-extension axis, but is unaffected by rotation about the long-axis (see animations provided in supplementary material).

**Joint angle conventions**

The conventions for joint angle measurements used in the previous studies of the gorilla muscle moment arms (Payne et al. 2006b) and kinematic studies (Isler 2005; Watson et al. 2009) were converted to the system used in our GaitSym model, as shown in Figure S1 below. Images of our model with all joints shown in flexed positions can be found in Figure S2.


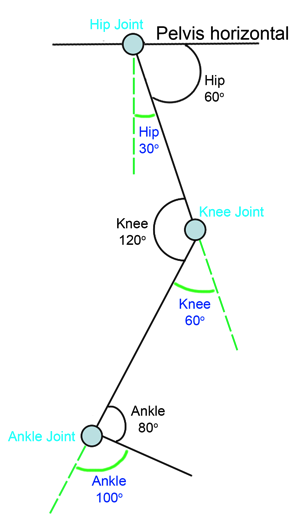


**Figure S1.** Conventions for joint angle measurements used in the previous studies of *Gorilla* muscle moment arms (Payne et al. 2006b) and limb kinematics (Isler 2005; Watson et al. 2009) compared to those used in our model. Values derived for the posture shown above using the convention used in our model are indicated by the green curves and blue angles/text, while those of previous studies are represented by the black curves and angles/text. The dashed green lines indicate a joint angle of zero (neither flexed nor extended) for each segment in the convention used in our model. All joint angle values and ranges from past studies were converted to the convention used in our model and shown above for the purpose of the comparisons made in the main text.

**
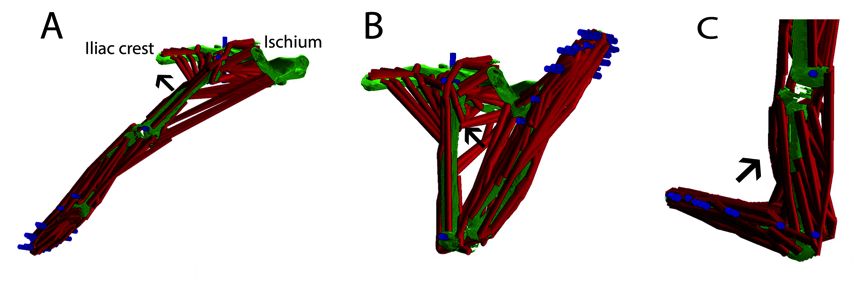
**

**Figure S2.** (A) Hip, (B) knee and (C) ankle in flexion. Black arrows show direction of flexion.

**Hip adduction-adduction angles**

To provide the first insight into the effect of 3D limb position on muscle moments in the gorilla, and to extend our assessment of locomotor optimality into 3D, we also generated moment arms across a range of flexion-extension angles measured in gorilla with the hip abducted at 0°, 30° and 50°, and adducted at 20° (Figure S3), which covers the majority of the kinematic ranges used during climbing, terrestrial quadrupedalism and bipedal walking ([Isler 2005](#_ENREF_36); [Watson et al. 2009](#_ENREF_75)).


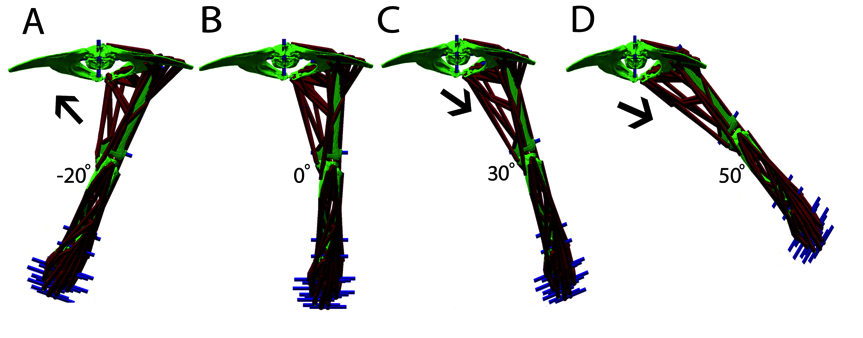


**Figure S3.** From left to right, hip at (A) -20°, (B) 0°, (C) 30° and (D) 50° abduction. Black arrow to the left shows direction of adduction, black arrows to the right show direction of abduction.
